# Heterogeneity in semantic priming effect with a lexical decision task in patients after left hemisphere stroke

**DOI:** 10.1590/S1980-5764-2016DN1002004

**Published:** 2016

**Authors:** Candice Steffen Holderbaum, Letícia Lessa Mansur, Jerusa Fumagalli de Salles

**Affiliations:** 1 PhD in Psychology (UFRGS) - Post-Graduation Program in Psychology, Federal University of Rio Grande do Sul (UFRGS).; 2 PhD in Linguistics (University of São Paulo) - Associate Professor of the University of São Paulo.; 3 PhD in Psychology (UFRGS) - Associate Professor on the Post-Graduation Program in Psychology, Federal University of Rio Grande do Sul (UFRGS)

**Keywords:** semantic priming, stroke, case series, cognitive neuropsychology, aphasia

## Abstract

Investigations on the semantic priming effect (SPE) in patients after left hemisphere (LH) lesions have shown disparities that may be explained by the variability in performance found among patients. The aim of the present study was to verify the existence of subgroups of patients after LH stroke by searching for dissociations between performance on the lexical decision task based on the semantic priming paradigm and performance on direct memory, semantic association and language tasks. All 17 patients with LH lesions after stroke (ten non-fluent aphasics and seven non aphasics) were analyzed individually. Results indicated the presence of three groups of patients according to SPE: one exhibiting SPE at both stimulus onset asynchronies (SOAs), one with SPE only at long SOA, and another, larger group with no SPE.

## RESUMO

As investigações sobre o efeito de *priming* semântico (SPE) em pacientes após lesões cerebrais no hemisfério esquerdo (LH) têm mostrado divergências que podem ser explicadas pela variabilidade no desempenho encontrado entre os pacientes. O objetivo do presente estudo foi verificar a existência de subgrupos de pacientes pós-AVC no hemisfério esquerdo do cérebro, buscando associações e dissociações entre o desempenho na tarefa de decisão lexical no paradigma de priming semântico e o desempenho em tarefas diretas de memória, tarefas de associação semântica e de linguagem. Todos os 17 pacientes com lesão no LH após acidente vascular cerebral (dez afásicos não-fluentes e sete não afásicos) foram analisados individualmente. Os resultados indicaram a presença de três grupos de pacientes de acordo com o efeito de priming semântico. Um grupo de participantes que mostrou SPE em ambos os SOAs, um dos participantes com SPE apenas no SOA longo e um grande grupo sem SPE.

## INTRODUCTION

The semantic priming effect (SPE) can be understood as an improvement in performance derived from context, in which target processing is facilitated by the preceding stimulus (prime) because of a semantic association between them. The left hemisphere (LH) plays an important role in this phenomenon,^1^ but it is unclear whether patients with LH lesions have a similar SPE to controls. No previous studies evaluating non-aphasic patients with LH lesion were found.

Studies evaluating non-fluent aphasics are inconclusive.^2^ Some studies found the SPE in expressive aphasics^3^ ^-^ ^5^ whilst others did not.^6^ ^,^ ^7^

SPE findings in these patients were inconsistent, probably because of methodological differences among studies (number and characteristics of patients' lesions, task stimuli, stimulus onset asynchrony, and so on).^8^ With regard to non-fluent aphasia in particular, a previous review^2^ showed that even when controlling stimulus onset asynchrony (SOA) (an important source of variance in the SPE), results remained conflicting 

Moreover, for auditory pairwise lexical decision semantic priming studies, in which all of the investigations found significant overall semantic priming results in Broca's aphasia,^7^ performance proved inconsistent. Baum (1997) showed that although non-fluent aphasic patients presented similar patterns to controls in terms of semantic priming, only 50% of these patients (those with less severe deficits in semantic processing) showed the semantic priming effect.^9^

Holderbaum et al. (2014) compared patients who had suffered LH lesion due to stroke and healthy control subjects. Individualized analyses indicated large variability in performance among participants of the same group.^8^ Case series have previously been used in the investigation of semantic memory and semantic processing.^10^ ^,^ ^11^ However, excluding group studies, only single cases assessing the SPE in patients with LH lesions were found.^4^ ^,^ ^5^ ^,^ ^12^

The only case series study of the SPE available assessed patients with right hemisphere lesions.^13^ Despite the SPE detected on the group analyses with an SOA of 500ms, and similar performance between clinical and control groups, when patients were investigated individually, data showed great variability among patients.^13^ For instance, on individual analyses, 25% of patients exhibited no SPE while 30% had impaired performance on direct lexical-semantic tasks such as verbal fluency with semantic criteria.

The aim of the present study was to investigate the existence of subgroups of patients after LH stroke by searching for dissociation between performance on the lexical decision task based on the semantic priming paradigm and performance on direct memory and language tasks.

## METHOD

Participants. The sample comprised 17 patients who had suffered a stroke to the left hemisphere, selected from two Brazilian public hospitals. Patients consisted of eight males and nine females with ages ranging between 33 and 73 years (mean = 57.9; standard deviation (SD) = 10.4). Of these patients, ten were expressive aphasics and seven were non-aphasics according to speech therapist evaluations. Around 75% of the participants were currently undergoing, or had already received, language rehabilitation. Detailed information about participants is given in Table 1, as previously published in a comparison group study involving the same sample.^8^

**Table 1:** Detailed information about participants^8^.

Patient	Age (years)	Years of education	Economic class	Stroke	Months post-onset	LH local lesion	Aphasia
MP1	63	10	C1	H	126	T	T. Motor
MP 2	70	7	B2	I	45	F	T. Motor
MP3	53	5	B2	I	125	FTP	Broca
MP 4	66	8	C2	I	8	FT	Broca
MP5	49	11	C1	H	36	T	T. Motor
MP6	54	8	C1	H	2	BG	Non-aphasic
MP7	52	16	A2	I	18	CR	Non-aphasic
MP8	60	15	B2	I	36	IC	Non-aphasic
FP1	33	15	C1	I	40	Unknown	T. Motor
FP 2	46	9	C1	I	70	FT	Broca
FP 3	48	15	C2	I	60	FTP	Broca
FP4	73	4	C2	I	24	PO	Non-aphasic
FP5	70	4	C1	I	7	TH	Non-aphasic
FP 6	63	4	B2	I	69	TP	Broca
FP7	58	5	C1	H	29	BG	Non-aphasic
FP 8	66	4	C2	I	120	FTP	Broca
FP 9	61	9	C1	I	45	TH	Non-aphasic

MP: male patient; FP: female patient; H: hemorrhagic stroke; I: ischemic stroke; T: temporal; F: frontal; P: parietal; O: occipital; BG: basal ganglia; CR: corona radiata; IC: internal capsule; TH: thalamus; T. Motor : transcortical motor

The following inclusion criteria were adopted: a minimum of four years´ regular education (mean = 8.2, SD = 4.1), right manual dominance, Brazilian Portuguese as native language and absence of evidence of depression (measured by the Beck Depression Inventory - BDI^14^ ^,^ ^15^ or Geriatric Depression Scale - GDS^16^ ^,^ ^17^), left hemisphere unilateral stroke, confirmed by neuroimaging exams; stroke at least 2 months before the assessment; and absence of any other neurological diseases. Patients were divided into two categories: non-aphasics and expressive aphasics, according to language abilities (Boston Diagnostic Aphasia Examination - BDAE short version;^18^ ^-^ ^20^ Token Test^21^ ^,^ ^22^). 

Procedures. The present study was approved by the Research Ethics Committees of the Psychology Institute of the Federal University of Rio Grande do Sul. In general, three sessions were needed to apply all the procedures below. A case series study was developed in order to find differences and similarities among participants´ performance.^23^ Impairment in performance on the Pyramids and Palm Trees Test and on the verbal memory, oral reading, reading comprehension and verbal fluency tasks of the NEUPSILIN-Af were identified as scores ≤ -1.5 SD,^24^ considering the normative data.

Pyramids and Palm Trees - three figures version,^25^ this was applied to assess explicit semantic processing. 

Instrument of Brief Neuropsycholinguistic Evaluation for Expressive Aphasics - NEUPSILIN-AF,^26^ this instrument examines temporal and spatial orientation, attention, perception, arithmetic abilities, language, memory, praxis and executive functions. 

Lexical decision task in a semantic priming paradigm:^27^ entails presentation of pairs of stimuli on a computer screen. The first stimulus of each pair (prime) was a real word whilst the second one (target) could be either a word or a pseudoword. The 95 word targets had up to seven letters, most of them being concrete or abstract nouns plus some adjectives and adverbs. Two equivalent versions of the task were developed. In order to ensure equivalence of versions, they had similar association strength between prime and target words and similar frequency and length of targets. Considering the SOA, each version of the experiment was conceived at two different SOAs (300ms - automatic processes - and 500ms - strategic processes). The time line of the experiment was as follows: prime appeared in lowercase letters for 300ms. In the case of 300ms SOA, the target was presented immediately after the prime, whereas for the 500ms SOA experiment, a distracter (+) was presented for 200ms between prime and target. In both cases, the target remained on screen for 3000ms. 

The experiment was presented using an E-prime computer program, which also recorded the answers and latencies. Stimuli were displayed at the center of the screen, in black letters (font Arial 24) against a white background. Primes appeared in lowercase letters while targets were shown in uppercase. 

Participants were tested individually, in a quiet room, seated approximately 60 cm from the screen. They were asked to rest the fingers of their left hand on two buttons of the keyboard and answer "YES" if the target was a real word (pressing key "1" on the keyboard) and "NO" if it was a pseudoword (pressing key "3"on the keyboard). The decision to use the left hand was made based on the fact that several patients had motor disabilities to the right side of the body as a consequence of LH stroke.^3^ ^,^ ^5^ ^,^ ^7^ Because all participants performed the task with both SOAs, the tasks were administered in two sessions. Each session lasted about 17 minutes for each participant. In order to prevent participants from seeing the exact same task twice, they performed one task from version one and the other from version 2.

## RESULTS

Regarding the SPE, reaction times (RT) for related and unrelated conditions of the lexical decision task (of each participant) were compared in order to investigate differences. The SPE was considered existent in cases of statistically significant differences between one condition and the other, as measured by the t-test for independent samples. It should be noted that accuracy is another possible variable for analysis in lexical decision task studies but was not the focus of the present investigation. Nevertheless, group analysis showed accuracy of between 75% and 95% depending on the target (word or pseudoword).^8^ Table 2 shows the performance of each participant on these tasks. 

**Table 2:** Performance of participants on SPE, Pyramids and Palm Trees Test, verbal memory, oral reading task and reading comprehension task of the NEUPSILIN-Af.

Patient	Magni SPE 300ms	Magni SPE 500ms	Palm tree (z-score)	Ver Mem (z-score)	Oral reading (z-score)	Reading comp (z-score)
MP1	-7 (.89)	116 (>.01)*	49 (.63 SD)	26 (1.8 SD)	11 (-2.5 SD)**	3 (.40 SD)
MP2	47 (.07)	11 (.70)	50 (.97 SD)	21 (.78 SD)	7 (-5.2 SD)**	3 (.45 SD)
MP3	29 (.70)	305 (>.01)*	41 (-2.2 SD)**	21 (.79 SD)	4 (-9.9 SD)**	3 (.47 SD)
MP 4	41 (.22)	107 (.17)	49 (.63 SD)	23 (1.2 SD)	12 (.58 SD)	3 (.45 SD)
MP5	322 (>.01)*	410 (>.01)*	38 (-3.2 SD)**	23 (.30 SD)	11 (-2.7 SD)**	2 (-3.2 SD)**
MP6	6 (.90)	-14 (.78)	47 (-.04 SD)	17 (-.16 SD)	12 (.56 SD)	3 (.47 SD)
MP7	24 (.66)	-23 (.64)	50 (.97 SD)	33 (2.4 SD)	12 (.37 SD)	3 (.32 SD)
MP8	11 (.79)	72 (.36)	45 (-.74 SD)	17 (-.52 SD)	12 (.30 SD)	3 (.40 SD)
FP1	NT	-70 (.43)	49 (.63 SD)	14 (-1.7 SD)**	10 (-6.1 SD)**	2 (-5.0 SD)**
FP 2	-40 (.49)	-64 (.40)	51 (1.3 SD)	20 (-.37 SD)	5 (-21 SD)**	3 (.32 SD)
FP 3	41 (.55)	-77 (.45)	44 (-1.1 SD)	25 (.68 SD)	11 (-2.7 SD)**	3 (.32 SD)
FP4	19 (.86)	27 (.81)	46 (-.39 SD)	26 (.89 SD)	10 (-5.8 SD)**	3 (.66 SD)
FP5	105 (.24)	-20 (.84)	47 (-.04 SD)	17 (.44 SD)	12 (.70 SD)	2 (-.88 SD)
FP 6	NT	366 (.10)	NT	16 (.14 SD)	0 (-4.5 SD)**	2 (-.88 SD)
FP7	-103 (.24)	-19 (.86)	41 (-2.2 SD)**	20 (.55 SD)	7 (-6.0 SD)**	2 (-1.4 SD)
FP 8	100 (.10)	137 (.07)	48 (.31 SD)	17 (.44 SD)	11 (.27 SD)	3 (.66 SD)
FP 9	63 (.32)	67 (.25)	37 (-3.5 SD)**	11 (-1.7 SD)**	0 (-33 SD)**	1 (-5.3 SD)**

MP: male patient; FP: female patient; NT: not tested; Magni SPE 300ms: magnitude of SPE at 300ms SOA; Magni SPE 500ms: magnitude of SPE at 500ms SOA; Palm tree: total score on the Pyramids and Palm Trees Test; Ver Mem: total score on the verbal memory tasks of the NEUPSILIN-Af; Oral reading: score on the oral reading task of the NEUPSILIN-Af; Reading comp: score on the reading comprehension task of the NEUPSILIN-Af. *indicates statistical significant SPE; **indicates impaired performance (≤ -1.5 SD).

## DISCUSSION

Three groups of participants were discriminated according to the SPE. The first group was formed by participants that presented no SPE. These 14 cases represented most of the participants, half of whom were aphasic and the other half non-aphasic. 

The second group consisted exclusively of participant MP5, who was the only participant to have shown significant SPE at both short and long SOAs. At 49 years old, MP5 was the second-youngest participant in the sample, and had eleven years of formal education. He was an expressive aphasic after having suffered a hemorrhagic stroke that caused a lesion to his temporal lobe. He performed poorly on most direct tasks analyzed but also on verbal memory. More specifically, the difficulty on reading tasks may explain why the semantic-related context helped improve his speed and accuracy on the lexical decision task (300ms SOA - accuracy of 100% for related trials, 58% for unrelated trials and 92% for pseudowords; 500ms SOA - accuracy of 93% for related trials, 17% for unrelated trials and 93% for pseudowords). The results of the lexical decision task also proved the dissociation between direct and indirect performance on reading tasks (lexical access) since, despite reading impairments on the NEUPSILIN-Af, he showed preserved lexical access on the indirect evaluation. This participant also demonstrated dissociation between impaired direct performance (Pyramids and Palm Trees Test) and preserved indirect performance on semantic processing. 

The third group of cases comprised two expressive aphasic participants (MP1 and MP3) who showed a SPE at the long SOA. MP1 had suffered a hemorrhagic stroke that caused a lesion to his temporal lobe. He was 63 years old and had 10 years of formal education. In contrast to MP5, MP1 had good performance on all tasks except oral reading. The impaired oral reading was probably influenced by the expressive aphasia. The lexical decision task corroborated this, on which the patient had only a small number of incorrect answers, suggesting appropriate lexical access. The presence of the SPE only at the long SOA indicated the patient was able to benefit from context when given enough time to process the stimuli and achieve lexical access. 

MP3, on the other hand, was a 53-year old expressive aphasic who had suffered an ischemic stroke affecting frontal, temporal and parietal regions. Besides the word oral reading impairment also found in MP1, participant MP3 performed poorly on the direct evaluation of semantic association (Pyramids and Palm Trees Test), suggesting a dissociation between direct and indirect (lexical decision task in the semantic priming paradigm) evaluation of this evaluation. One characteristic that distinguished the two participants from others not presenting the SPE, was the number of months post onset, that indicated at least ten years after the stroke in each case. 

There was, however, one participant that, although not exhibiting a statistically significant SPE on RT analysis, showed strong evidence of a major SPE. FP6 had a high magnitude of SPE at the long SOA, responding on the related trials 366ms faster than for unrelated ones. Moreover, despite poor performance on the lexical decision task (accuracy of 72% for related trials, 45% for unrelated trials and 46% for pseudowords), her percentage of correct answers was higher for the related trials which suggested facilitation promoted by the semantic context. The patient was a 63- year-old woman with four years of formal education who had suffered an ischemic stroke causing a left temporo-parietal lesion. On the direct task, she performed within the norms expected for her age and educational level. Akin to the previous participants mentioned above, the Broca's aphasia in this patient probably explained the poor performance on the oral reading task. Considering the evidence of SPE, this could be understood as an association between indirect and direct evaluations of memory, semantic processing and reading. 

A double dissociation was found in the comparison among participants with preserved SPE and impaired performance on the Pyramids and Palm Trees Test (MP3 and MP5) and participants with impaired SPE and preserved performance on the Pyramids and Palm Trees Test (MP2, MP4, MP6, MP7, MP8, FP1, FP2, FP3, FP4, FP5, FP8). Dissociative performance was found in most cases with impaired SPE yet normal scores on memory direct evaluation (MP2, MP4, MP6, MP7, MP8, FP2, FP3, FP4, FP5, FP6, FP7, FP8). 

In terms of reading abilities, a double dissociation was found between direct (oral reading) and indirect (lexical decision task in the semantic priming paradigm) evaluation. Primarily, cases showed impaired SPE and preserved oral reading (MP4, MP6, MP7, MP8, FP5, FP8). However, there was also one case that demonstrated the opposite pattern, with preserved SPE at both SOAs and impaired performance on the oral reading task (MP1, MP3 and MP5). Double dissociation was indicated by the presence of impaired reading comprehension and preserved SPE (MP5) versus preserved reading comprehension and impaired SPE (MP2, MP4, MP6, MP7, MP8, FP2, FP3, FP4, FP5, FP6, FP7, FP8).

The data provided evidence of large heterogeneity among cases, where this variability was found not only on the lexical decision task in the semantic priming paradigm but also on the direct tasks of memory, semantic association and language. This could be seen, for instance, in the several double dissociations found between direct and indirect tasks. Our data has shown different groups of cases according to cognitive performance. 

Results showed a small number of participants with SPE, as indicated by t-tests, although a large number of subjects demonstrated a positive magnitude for SPE. This finding is congruent for the short SOA, if the analysis data of the group as a whole is considered, which indicated absence of SPE at this SOA.^8^ Nonetheless, this group analyses revealed the presence of SPE at the long SOA,^8^ whereas individual analyses suggested that only 3 of the 17 participants had statically significant SPE.

Data from a previous study^27^ were reanalyzed. The study in question compared SPE between a group of third graders and a group of college students (all normal readers and neurologically healthy) using a short (250ms) and a long (500ms) SOA. The SPE was found in both groups for the two SOAs^27^ but only group analyses were performed . Individual t-tests for independent sample were carried out for all adults and detected a SPE in 26% of subjects at 250ms SOA and in 34% at 500ms SOA.

The finding of approximately one third of significant SPE on individual analyses when the SPE is considered present for the sample as a whole on group analyses might be explained by statistical power. Because of the small effect size typically found in social psychological effects, the sample size should be at least 90 for a statistical power for independent sample t-tests ≥ .90.^28^ However, such a large sample size is rarely achievable in studies.^28^ In the present experiment, for example, t-tests were performed with a mean sample size of 47 items under each condition, which is probably insufficient to detect the presence of the SPE in a single case. The first alternative would be to increase the sample size, although this is often difficult to accomplish for two reasons: increasing the number of participants is not easy because of the difficulty in recruiting participants, especially with all the inclusion and exclusion criteria. Another possibility would be to increase the number of items in the task but this would become excessive and exhausting, consequently interfering in the performance of the participants. Other than this, increasing the sample in an attempt to raise the statistical power of the study could be achieved by using confidence interval width to determine sample size, using equivalence testing, evaluating specific expectations as opposed to testing the null hypothesis, and by employing Bayesian approaches.^28^

That being said, it cannot be concluded that participants not presenting statistical SPE, did not benefit from the semantic context. Semantic priming phenomena may have been present and the statistical analyses not sensitive enough to detect this difference between related and unrelated conditions. 

The present study has shown that the variability in performance among patients exists even when considering the dissociation and association between direct and indirect tasks. All functions evaluated - memory, semantic association and language - involved participants with both types of performance, thereby leading to several double dissociations. Finally, it is important to highlight that all participants who showed statistically significant SPE were aphasics. Moreover, in the group of participants that showed no SPE, there were no differences in performance between aphasics and non-aphasics except on the oral reading task. This proves that evaluating aphasic patients with adapted tasks^26^ allows proper assessment of cognitive functions, showing that differences in performance between aphasics and non-aphasics are a consequence of language disability. It also showed that the lexical decision task is appropriate for evaluating this type of population because it does not require verbal answers. Nevertheless, it is important to note that about 75% of the participants were undergoing, or had received, language rehabilitation, and that this likely biased their performance. In addition, the heterogeneity in terms of lesion location is another variable that should be taken into account to explain the disparate cognitive performances in the sample assessed.

Case series investigations preserve individual data^23^ instead of removing variability as group studies do. For this reason, the heterogeneity found so frequently becomes accepted, and even necessary, with covariation in patients´ performance between tests providing the basis for scientific inference.^23^ The results reported provide information for clinical neuropsychology about cognitive functioning in patients who have suffered stroke to the LH. 

The heterogeneity of performance in such cases should be taken into account for evaluation and rehabilitation decisions. Furthermore, the presence of association and dissociation between direct and indirect tasks should prompt neuropsychologists to use more than one type of task to evaluate each function. Lastly, this association and dissociation can play an important role in deciding how to compensate for impairments. Further studies should be conducted to increase the number of trials and statistical power for detecting the SPE or to search for more reliable statistical analysis to infer the presence or absence of SPE in individual analyses. 
